# The Sixth Rhino: A Taxonomic Re-Assessment of the Critically Endangered Northern White Rhinoceros

**DOI:** 10.1371/journal.pone.0009703

**Published:** 2010-04-07

**Authors:** Colin P. Groves, Prithiviraj Fernando, Jan Robovský

**Affiliations:** 1 School of Archaeology & Anthropology, Australian National University, Canberra, Australian Capital Territory, Australia; 2 Centre for Conservation and Research, Rajagiriya, Sri Lanka; 3 Department of Zoology, Faculty of Science, University of South Bohemia, České Budějovice, Czech Republic; American Museum of Natural History, United States of America

## Abstract

**Background:**

The two forms of white rhinoceros; northern and southern, have had contrasting conservation histories. The Northern form, once fairly numerous is now critically endangered, while the southern form has recovered from a few individuals to a population of a few thousand. Since their last taxonomic assessment over three decades ago, new material and analytical techniques have become available, necessitating a review of available information and re-assessment of the taxonomy.

**Results:**

Dental morphology and cranial anatomy clearly diagnosed the southern and northern forms. The differentiation was well supported by dental metrics, cranial growth and craniometry, and corresponded with differences in post-cranial skeleton, external measurements and external features. No distinctive differences were found in the limited descriptions of their behavior and ecology. Fossil history indicated the antiquity of the genus, dating back at least to early Pliocene and evolution into a number of diagnosable forms. The fossil skulls examined fell outside the two extant forms in the craniometric analysis. Genetic divergence between the two forms was consistent across both nuclear and mitochondrial genomes, and indicated a separation of over a million years.

**Conclusions:**

On re-assessing the taxonomy of the two forms we find them to be morphologically and genetically distinct, warranting the recognition of the taxa formerly designated as subspecies; *Ceratotherium simum simum* the southern form and *Ceratotherium simum cottoni* the northern form, as two distinct species *Ceratotherium simum* and *Ceratotherium cottoni* respectively. The recognition of the northern form as a distinct species has profound implications for its conservation.

## Introduction

As much a cause for celebration the conservation success of the Southern white rhino is, equally shocking and dire is the fate of the Northern white rhino. After recovering from a handful of survivors at the turn of the 20^th^ century, the Southern form escaped relatively unscathed from the large-scale African rhino poaching epidemic of the 1980s. In contrast, the once tolerably numerous Northern form has been reduced to a tiny remnant (less than 20) in the Garamba National Park, Democratic Republic of Congo, and a similar number in two zoos. Teetering on the brink of extinction, its in-situ and ex-situ survival hang by a thread. Urgent and concerted effort is required to stave off its extinction. The taxonomic status of the Northern form is central to determining its conservation importance and will be a critical driver of efforts to save it.

In the thirty years since the last taxonomic revision of the White Rhinoceros, genus *Ceratotherium* [1], new material and analytical tools have become available, necessitating a reassessment of the taxon. The metrical data of Groves [1], and some collected subsequently, can be re-analysed using sophisticated statistical packages that have become more readily available. Detailed information and measurements have been published on a remarkable Early Pleistocene skull KNM-ER 328C [2]; this had earlier been reported briefly by Hooijer [3]. Further material and analysis has been published by Guérin [4]–[7]. The external phenotypic differences between Northern and Southern forms of White Rhino tentatively raised by Groves [8] have been extended and supplemented by Hillman-Smith and colleagues [9], [10]. The reality of these distinctions needs to be examined.

Genetics has become an important criterion in establishing taxonomic identity. The chromosomes of northern and southern white rhinos apparently do not differ consistently; the typical diploid number is 82, but a northern male had 2n = 81 (heterozygous for a Robertsonian translocation) as did his two female offspring [11]. Merenlender et al. [12] found electrophoretic variation on 25 allozyme loci between northern and southern white rhinos to be unexpectedly low: Nei's distance was 0.005, compared with a distance of 0.32 between *Ceratotherium* and *Diceros*. Estimates of heterozygosity were low for all rhino taxa examined in their study and less than 0.1% of loci were polymorphic in any of the three taxa. Stratil et al. [13] studied some of the same individuals of northern white rhinos and found much greater protein polymorphism, which they attributed to the use of more sophisticated and sensitive methods. George et al. [14] found a fixed difference in serum esterase between the two white rhino taxa – ES3 being present in all southern white rhinos (n = 23), but in none of the northern (n = 7). George et al. [15], using restriction enzyme analysis of mtDNA on a single individual of each white rhino subspecies, found 4% difference, compared to about 7% between white and black rhinos. A subsequent study by some of the same authors, with a higher sample of individuals and additional restriction enzymes, estimated the mtDNA divergence between the two white rhinos at 1.4%, and the inter-generic divergence at 4.5% [14]. Morales and Melnick [16], based on restriction enzyme analysis of a 1.6 kb mitochondrial ribosomal gene segment, found 0.3% sequence divergence between the white rhinos, and 1.8–2.1% between white and black rhinos. Thus, previous genetic analyses have provided conflicting results on the divergence between the two white rhino taxa.

Here we report on a reassessment of the taxonomic status of the white rhinos based on new material and reanalysis of existing data, and review ancillary information on the taxa.

## Results

### Dentition

In all skulls of *Ceratotherium simum simum* examined, the protoloph on the molars and the posterior premolar, sweeps backward from about one third of its length, so that it runs more distally than lingually for the remaining two thirds. In all *C. s. cottoni*, about one half or more of the protoloph is distolingual in direction.

In the southern form, the ectoloph on the third molar is produced back more behind the metaloph, to form a larger metastyle.

### Dental Metrics

Measurements of mean crown heights taken by CPG in skulls of southern white rhinos varied in both M1 and M2 from 45 to 72 (n = 4 for both teeth), and in northern skulls the range was 35–52 (n = 10 M1, n = 7 M2).

### Cranial Anatomy

The palate ends approximately level with the junction of the second and third molars in the southern form, and halfway along the second molar in the northern.

The incisive foramen ends level with about three quarters of the way along the second premolar in the southern form, and level with the anterior edge of, or one quarter of the way along, the second premolar in the northern.

### Cranial Growth

**Figure 1:**
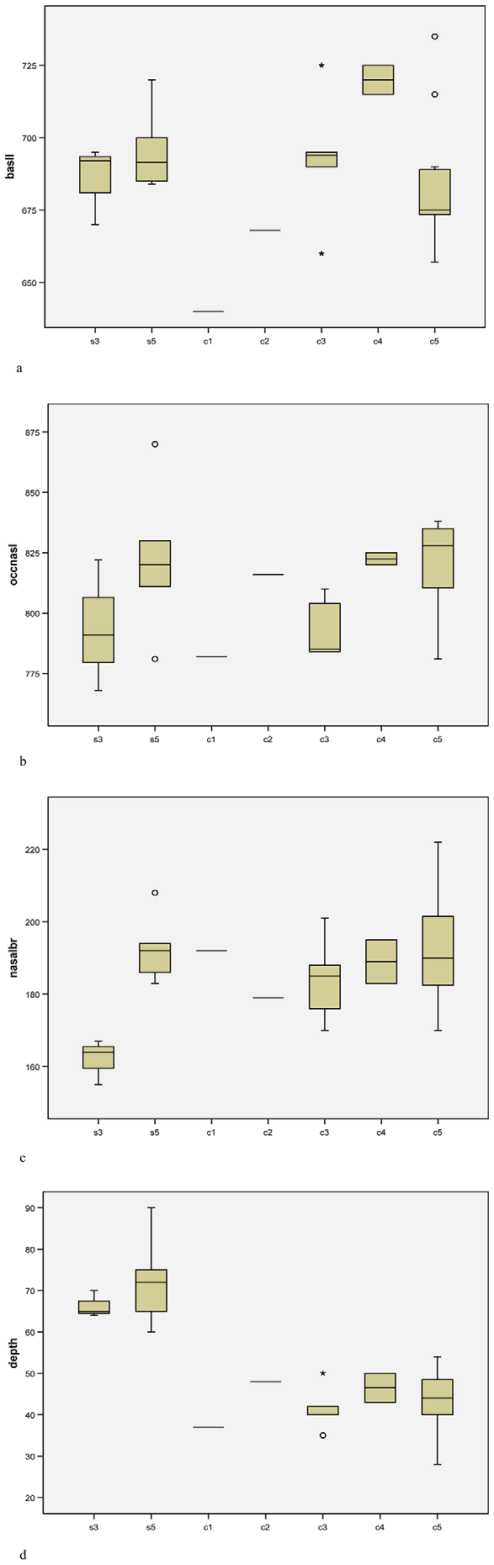
Skull growth in males: a, basal length; b, occipitonasal length; c, nasal boss breadth; d, depth of dorsal concavity. Age stages are as follows: Stage 2, first molar in process or erupting; 3, second molar in process of erupting, second and third premolars in process of replacement; 4, second molar in wear; fourth premolar in process of replacement; 5, third molar in process of eruption; 6, third molar in occlusion.

**Figure 2:**
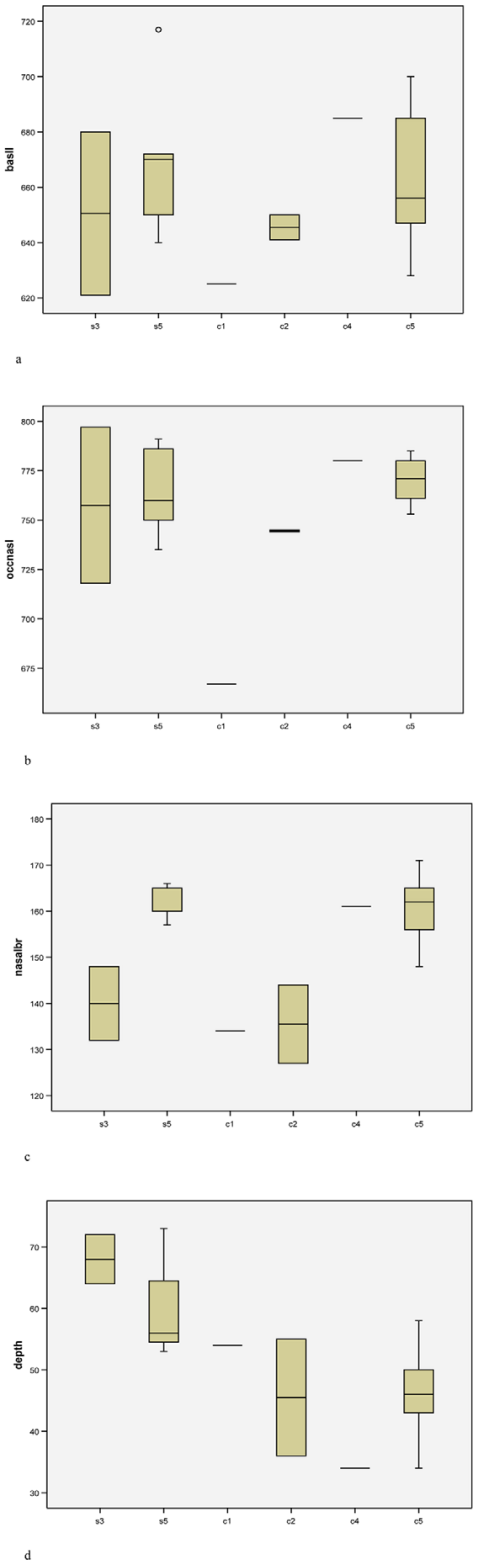
Skull growth in females: a, basal length; b, occipitonasal length; c, nasal boss breadth; d, depth of dorsal concavity. Age stages as in Figure 1.

Figure 1 depicts skull growth in males; Figure 2 in females. Basal skull length appears not to increase after stage 3 in males of *cottoni* (Figure 1a); there are no stage 3 skulls of females for *cottoni*, but certainly there is no difference between stages 4 and 5 (Figure 2a). There appears, on the other hand, to be some marginal increase in growth after stage 3 in both sexes of *simum* (Figures 1a, 2a). By contrast, in occipitonasal length, males of stage 3 are by no means full-sized in either taxon (Figure 1b), nor is one of the two available females of *simum* (Figure 2b).

Nasal breadth (Figures 1c, 2c) continues to grow noticeably between stages 3 and 4. In stage 3, the nasal boss of *simum* is narrower than that of *cottoni*, but the difference has disappeared by maturity. Even by stage 3, the male already has a wider nasal boss than the female, and the single stage 2 skull of male *cottoni* has wider nasals than the two corresponding stage females.

The depth of the dorsal concavity appears not to change with age in *cottoni* or in males of *simum* (Figures 1d, 2d), but the limited evidence suggests that the depth may decrease somewhat between stages 3 and 5 in *simum* females (Figure 2d).

**Figure 3:**
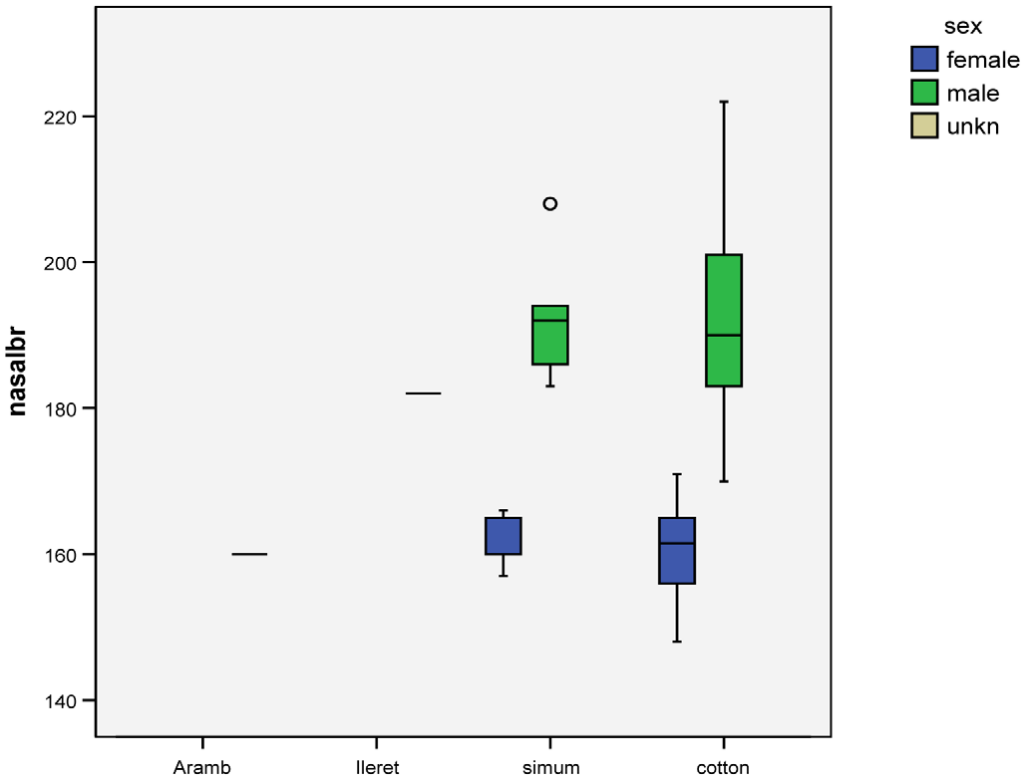
Nasal boss breadth.

Because there is no evidence for any difference between stages 4 and 5 in nasal boss breadth, these two stages have been combined in Figure 3. In the two living taxa, the values for the two sexes of *cottoni* just overlap, while those for *simum* (smaller sample) do not. This character can therefore be used with nearly complete confidence to allocate skulls whose sex is unknown. Nasal breadth measurements are available for the North African Arambourg skull and for the skull from Ileret. If these are comparable to modern white rhinos, the Arambourg skull will be a female, the Ileret skull probably a male.

### Cranial Metrics

**Figure 4:**
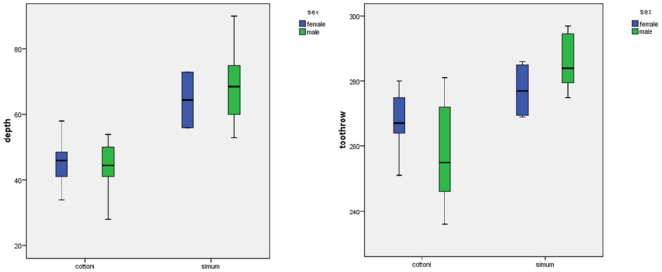
Univariate comparisons of living samples, stages 5–6: a, depth of dorsal concavity; b, maxillary toothrow length.

**Figure 5:**
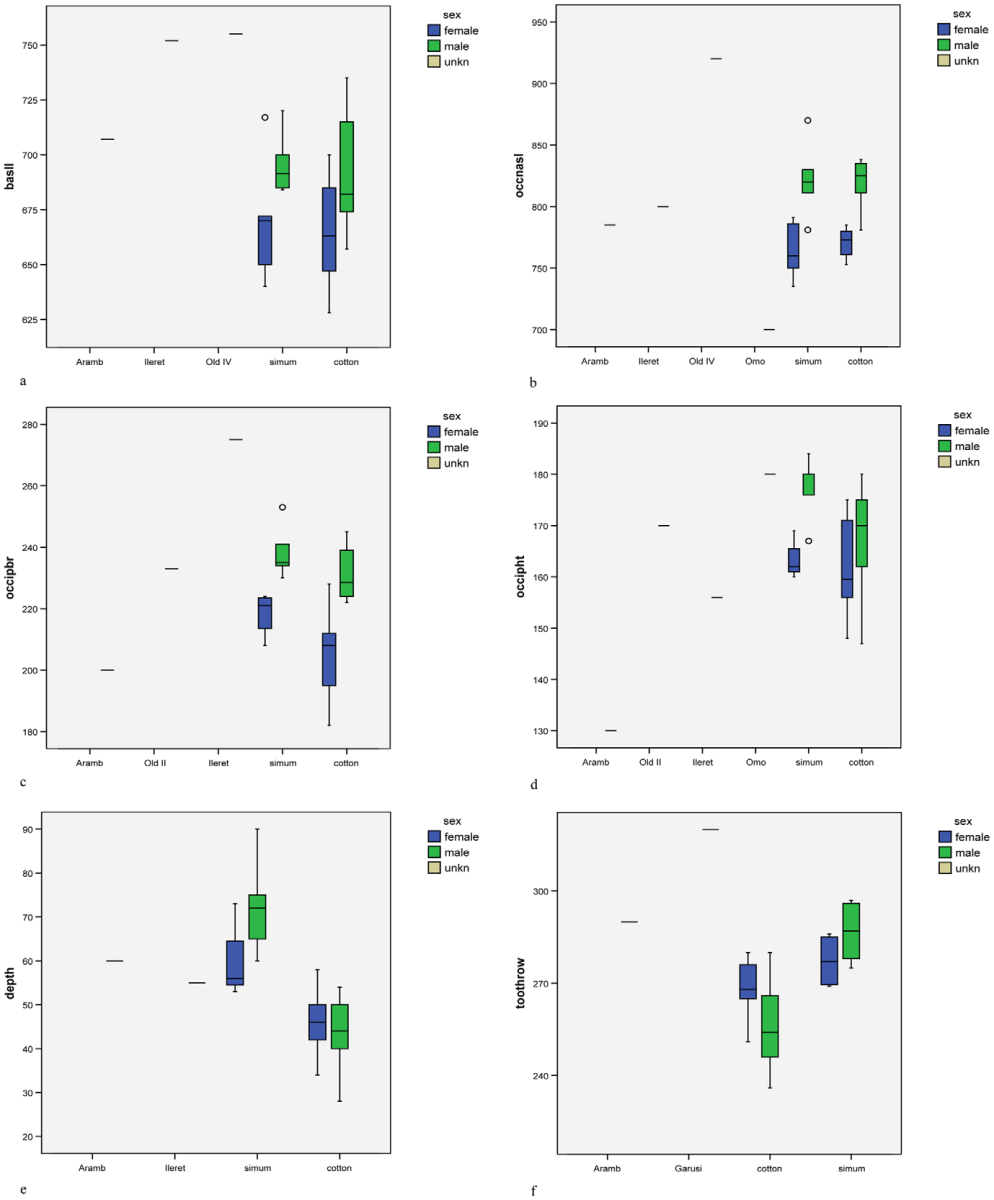
Univariate comparisons of living and fossil samples; a, basal length; b, occipitonasal length; c, occipital crest breadth; d, occipital height; e, depth of dorsal concavity; f, maxillary toothrow length.

Univariate comparisons between fully grown samples of living white rhinos are shown in Figure 4, and comparisons with fossil specimens in Figure 5.

Figure 4a shows the depth of the dorsal concavity in adults and 4b, maxillary toothrow length.

**Figure 6:**
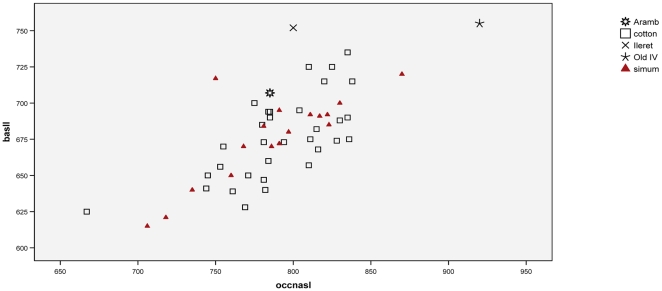
Bivariate plot: a, occipitonasal length relative to basal length; b, occipital height relative to occipital crest breadth; c, dorsal concavity relative to occipitonasal length.

Figure 5 continues the comparisons between the two living taxa, and extends them to those individual fossil specimens which are complete enough to take the measurements concerned. Figure 5a shows the basal lengths of living and fossil white rhinos and Figure 5b occipitonasal lengths. Occipital breadth is shown in Figure 5c and Occipital height in Figure 5d. Figures 5e and 5f; depict depth of dorsal concavity and maxillary toothrow length, respectively. Figure 6 represents bivariate scatterplots for some of the skull measurements: occipitonasal length relative to basal length, occipital height relative to occipital crest breadth, and dorsal concavity relative to occipitonasal length. Insepction of actual skulls demonstrates that the difference in the depth of the dorsal concavity is easily detected visually.

**Figure 7:**
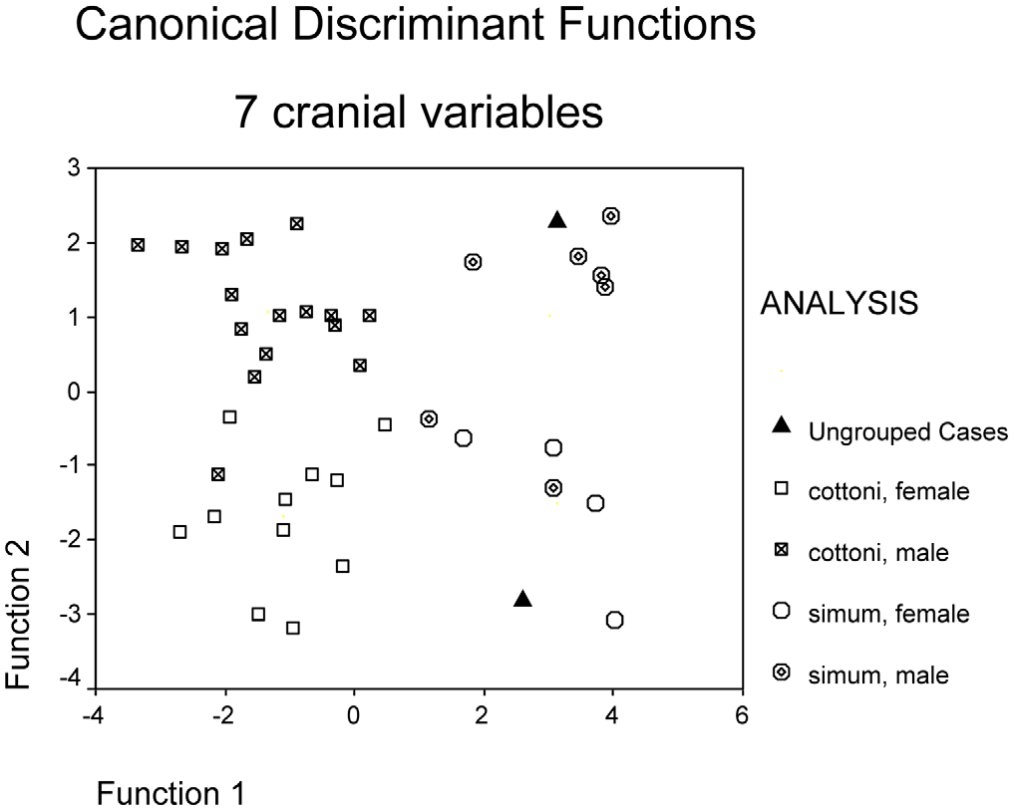
Discriminant analysis using 7 variables. Ungrouped fossil specimens, with their DF values, are: Ileret (3.12, 2.28), with *simum* males; Arambourg (2.59, −2.81), with *simum* females. DF1 accounts for 68.4% of total variance, DF2 for 31.1%.

Figure 7 is a scatterplot of the first two Functions of a Discriminant Analysis using 7 cranial variables: Occipitonasal length, Basal length, Zygomatic breadth, Occipital breadth, Occipital height, Nasal breadth and Dorsal concavity depth. Four groups were entered: southern males and females, and northern males and females; the Arambourg and Ileret fossils were entered as unknowns, meaning that they will be allocated to a position in the dispersion calculated on the basis of the four groups, but do not have a chance to extend the dispersion on their own account.

### Postcranial Skeleton

The crural index (tibia expressed as a percentage of femur length) measured 71–72% in three southern and 73–75% in three Northern rhino specimens.

### External Measurements

The heights of two Southern males at Zoo Usti nad Labem, Czech Republic, measured 168 and 165 cm; a female, 157 cm. All three were born in Umfolosi, 1966–1970. The height of a Northern female at Dvur Kralove (Najin) was 157 cm. Spine length of a Northern female (Najin) was 269 cm, of a male (Suni) was 271 cm; and that of a hybrid × Southern female (Nasi) was 269 cm.

### Genetics

The number of bps used for analysis from each fragment is given in Table 1. Analyzed segments showed consistent divergence between the two forms. No nucleotide polymorphisms were observed within *C. s. simum* or black rhino subspecies *D. b. minor* and *D. b. michaeli* in the analyzed mitochondrial 12S and NADH segments, or in the nuclear Amelogenin segment. The mitochondrial D-loop segment showed polymorphism within the two black rhino subspecies but not within *C. s. simum*. Divergences observed between the taxa in respect of each analysed segment are given in Table 1.

**Table 1:** Observed Sequence Divergence (Uncorrected p) in the Analyzed Fragments.

Fragment	Within subspecies			Between subspecies		Inter-generic	Subsp. divergence as % inter-generic		
	*C. s. simum*	*D. b. michaeli*	*D. b. minor*	*simum* vs *cottoni*	*michaeli* vs *minor*	*Ceratotherium* vs *Diceros*	*C. simum*	*D. bicornis*	Ratio *C.s./D.b.*
Amel X	0.0	0.0	0.0	0.2	0.1	0.8–0.9	23.5	11.7	2.01
12S	0.0	0.0	0.0	0.8	0.5	4.0–4.6	18.6	11.6	1.60
ND	0.0	0.0	0.0	1.3	0.7	8.0–8.5	15.8	8.5	1.86
D-loop	0.0	0.8	0.8	7.6	3.5–4.6	14.4–17.1	48.3	25.7	1.88

## Discussion

We found differences that were diagnostic of the two taxa in practically all characters examined.

### Dentition

The two forms of white rhino showed distinctive dental morphology. The protoloph on all molars and the posterior pre-molar in the southern form was oriented parallel to the toothrow in the distal two thirds, whereas it was diagonal for one half or more in the northern form. Additionally, the ectoloph on the third molar was produced back more behind the metaloph, forming a larger metastyle in the southern form. Therefore, dental morphology clearly distinguishes *simum* from *cottoni*.

### Dental Metrics

Guérin [5] suggested that the teeth are larger in southern white rhinos, especially (in the upper toothrow) P4, M1 and M2.

The index of hypsodonty is defined as:
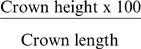
Guérin [5] gave hypsodonty values for two specimens of P4 in white rhinos as 188.68 and 201.96, contrasting with black rhinos at 134.65 and 142.20. For M3 he gave 136.76 in a white rhino, compared to 121.15 and 102.36 in two black rhinos. His white rhino indices would correspond to crown heights in the white rhinos of about 85 mm for M3, and 95 and 101 mm for P4. These compare well with the figures for M1 given by Hillman-Smith et al. [9] for southern whites of 88 at the time of eruption, rising to 97 at the time of the eruption of M2, and falling again thereafter as wear proceeds.

As remarked by Hillman-Smith et al. [9], true crown heights are difficult to measure on teeth still in place in the jaw, and crown height above the alveolar line is much easier to measure (if less anatomically exact), although because of continuing eruption the height remains constant for longer. Mean crown heights taken in this fashion on M1 remain at about 43–47 mm until quite an advanced stage of wear [9: Table IV]. Measurements taken by us indicate greater crown height in M1 and M2 in *simum* compared to *cottoni*, consistent with the presence of lower-crowned cheekteeth in northern white rhinos. The cheekteeth are thus lower-crowned as well as being somewhat smaller (see above).

### Cranial Anatomy

Differences in the morphology of the palate and the location of the incisive foramina showed diagnostic differences between the two forms. The palate was longer and the incisive foramen located more posteriorly in the southern form, than in the northern.

### Cranial Growth

A number of variations in cranial growth between the two forms were noted. The differences in the basal length and occipitonasal lengths of different stage skulls suggested the elongation of the occipital crest after stage 3 in both forms, but the differential growth of the occipital crest was greater in the northern form due to the earlier cessation of basal growth in *cottoni*. Although some differences in nasal breadth, suggesting a similar pattern to occipitonasal growth, were noted, the small sample sizes precluded identifying any fixed variation between the two forms.

### Cranial Metrics

The most striking sexual dimorphism, in a character nearly independent of body size, was shown by the width of the nasal boss. The difference between the sexes in this character was evident in both forms with the width usually being greater in males. This sexual dimorphism was accentuated in the case of the southern form with no overlap of measurements between the sexes.

Heller [17] concluded that northern and southern white rhinos differed by the depth of the dorsal concavity and by the length of the toothrow, and Groves [1] concurred. Our findings show that there is a striking difference between the two taxa: the depth is much greater in southern white rhinos, and the dorsal outline of the skull is very flat in northern. There is no overlap in males, but a slight overlap in females. In females the dorsal concavity appears to become slightly flatter with age, but not in males. Thus, Heller's claim of differences in dorsal concavity is borne out by our findings. Heller's [17] finding is vindicated for the maxillary toothrow as well (see above), although in this case there are overlaps in both sexes. There is no difference between the sexes in *cottoni* (the mean for females is somewhat larger than that for males, although within the quartile range), but females of the southern form do have a shorter toothrow than males.

Guérin [5: pp. 171–172] found that the skulls of the southern form were very slightly larger but the orbitotemporal fossa was longer, and, according to the text (p.171) that the occiput is wider in the northern, but his Table 42 (p.172) shows this to be the opposite (Guérin's measurement 16). Guérin also found the mandible of the southern white rhino was larger, with a longer symphysis; and the corpus and condyle higher. We can test the claim of a size difference and of a difference in the occipital crest, but we did not take any measurements of the mandible.

In comparing basal lengths, we found little or no difference between the two living taxa, but the basal lengths of the fossils from Ileret and Olduvai Bed IV were much greater than any living representative, and that of the Arambourg skull, which we suggested was female, was greater than any living female. The broad outlines were similar for Occipitonasal length, but with differences. The first difference was that one specimen from South Africa was considerably longer in occipitonasal length than any other, that is to say, it had an occipital crest that is posteriorly extended. The second difference was that the occipitonasal lengths of the Arambourg and Ileret skulls were not longer than modern females and males respectively, implying that the occiput was less posteriorly extended than in modern white rhinos.

We found that the occipital breadth was strongly sexually dimorphic, second only to nasal breadth, but in contrast to nasal breadth, the dimorphism in *cottoni* was greater. Between the northern and southern forms, occipital breadth measurements of males overlapped considerably, but those of females only very slightly, northern being much smaller than southern. So in this sense Guérin's findings are vindicated. All of the fossils that could be measured had an extremely broad occiput (if their sexes were correctly interpreted: see results). Thus it is clear that sexual dimorphism existed in the early Pleistocene, the Ileret skull having a broader occiput than modern males, the Arambourg skull broader than modern females. The occiput of the Olduvai Bed II specimen could be measured, and it was very broad like the Ileret specimen.

Occipital height was likewise highly sexually dimorphic, in this case more in the southern form than in the northern. In comparing the two forms, male southern skulls were almost absolutely larger in this measurement than male northern skulls. The measurement of the Arambourg skull was less than in any living specimen, while that of the Ileret skull was within the range of modern females; this supports the conclusion from occipitonasal length, that the occiput is less posteriorly extended (i.e. the occipital crest is shorter) in the fossil than in living specimens. The Olduvai Bed II specimen is somewhat larger in this measurement than the Ileret skull; the Kibish specimen is larger still, within the range of male southern skulls.

Of the two features used by Heller [17] to distinguish the two modern taxa, Arambourg and Ileret both have a relatively deep dorsal concavity like modern southern skulls (Figure 5e); the toothrow of the Arambourg skull is (assuming it is a female) longer than would be expected for a modern southern specimen, while that of the Garusi specimen is as long as would be expected were it a male of the same population (Figure 5f; the toothrow length of the Ileret skull is unavailable).

In bivariate analysis of skull measurements, some of the characters clearly separated the two extant taxa while others did not. The positioning of the fossil specimens was variable. In the occipitonasal length against basal length, there was as expected no difference between the two modern forms. The Olduvai Bed IV specimen, though it was very much bigger than any modern specimen, was modern in its proportions, the Arambourg skull was on the edge of modern proportions, while the Ileret skull was outside the modern range.

Occipital breadth to height comparisons confirmed the shorter occipital crest of the Arambourg and Ileret skulls, but the Olduvai Bed II specimen was within the modern range. When dorsal concavity depth was plotted against occipitonasal length (see above), the difference between the two modern taxa was striking, and there was no overlap although they come close; the Arambourg and Ileret skulls came just within the southern range.

In the discriminant function analysis using seven variables (Figure 7), the first Function (horizontal), which accounted for 68.4% of the total variance, was heavily weighted positively on dorsal concavity depth and, less heavily, on occipital breadth and occipitonasal length, and fairly strongly weighted negatively on nasal breadth and less strongly on occipital height (Table 2). The second Function, which accounted for 31.1%, was fairly heavily weighted positively on occipital nasal length and occipital breadth, less heavily on nasal breadth, and weakly negatively weighted on zygomatic breadth. Southern and Northern groupings separated completely in the discriminant function analysis, but the sexes within each group overlapped somewhat. The Arambourg skull fell within the dispersion of southern females, the Ileret skull within the southern males. All skulls were closer to the centroids of their own geographic groupings, which was also true of the leave-one-out cross- validations (Table 3). While a few males within each geographic sample could be misidentified as females, and vice versa, there was no misallocation of northern as southern or vice versa.

**Table 2:** Standardized Canonical Discriminant Function Coefficients for Figure 7.

	DF 1	DF 2
Occipitonasal length	0.311	0.562
Basal length	0.052	−0.010
Zygomatic breadth	0.041	−0.214
Occipital breadth	0.476	0.580
Occipital height	−0.269	0.099
Nasal breadth	−0.747	0.259
Dorsal concavity depth	1.083	0.072

**Table 3:** Classification Results for Figure 7.

Analaysis			Predicted Group Membership				Total
			*simum* m	*simum* f	*cottoni* m	*cottoni* f	
Original	Count	*simum* m	7	1	0	0	8
		*simum* f	0	5	0	0	5
		*cottoni* m	0	0	17	1	18
		*cottoni* f	0	0	1	13	14
		Ungrouped cases	1	1	0	0	2
	%	*simum* m	87.5	12.5	0	0	100.0
		*simum* f	0	100.0	0	0	100.0
		*cottoni* m	0	0	94.4	5.6	100.0
		*cottoni* f	0	0	7.1	92.9	100.0
		Ungrouped cases	50.0	50.0	0	0	100.0
Cross-validated	Count	*simum* m	6	2	0	0	8
		*simum* f	3	2	0	0	5
		*cottoni* m	0	0	16	2	18
		*cottoni* f	0	0	3	11	14
	%	*simum* m	75.0	25.0	0	0	100.0
		*simum* f	60.0	40.0	0	0	100.0
		*cottoni* m	0	0	88.9	11.1	100.0
		*cottoni* f	0	0	21.4	78.6	100.0

93.3% of original grouped cases correctly classified.

77.8% of cross-validated grouped cases correctly classified.

### Postcranial Skeleton

Guérin [5] found that the metapodials are a little bigger in *simum*, but his data show that in effect it is the medial ones that are slightly longer, the laterals being somewhat shorter. Of several measurements taken by CPG on postcranial bones, a difference appears only in the crural index, suggesting slightly longer limbs in *cottoni*.

### External Measurements

Hillman-Smith et al. [9] reported that full body size and sexual maturity in females are achieved at 6–8 years, but in males not until 10–15 years. They reported that adult males of southern white rhinos weigh 2000–2400 kg, and a subadult male, with the last molar not fully erupted, was already 2130 kg, and adult females weigh 1500–1700 kg. On the other hand, at 10–10½ years two northern males weighed only 1400 and 1600 kg and, at about the same age (9–10½ years), four northern females weighed 1400–1500 kg. Spine length (occiput to base of tail) was 259–284 cm in male and 248–273 cm in female southern white rhinos, and 266 cm in a northern adult (10-year-old) male and 245–262 cm in four northern females. To these may be added our measurements of 271 and 269 cm in a male and a female respectively, of the Northern form, just slightly larger than in Hillman-Smith et al.'s sample (the male, Suni, was also measured by Hillman Smith et al. [9], but when only three years of age). Shoulder height in two southern males Hillman-Smith et al.'s sample was 174–178 cm, and in two 10-year old northerns, 151–152 cm (virtually the same as in four 9–14-year-old females, 150–154 cm). Northern white rhinos, these authors remarked, ‘appear to be shorter and smaller’. Our own shoulder height data (Southern male 168 cm, females 157 and 165 cm; Northern female 157 cm) are comparable, though again, most of them are on the large side.

Other measurements of southern white rhinos exist. Kirby [18] gave the measurements of a male and a female, stated to be ‘large’, as 179 and 177 cm respectively. Hitchins (personal communication in [9]) gave two males as 178 and 174 cm.

Perhaps the largest series of body measurements in the literature for both forms is that of Heller [17]. (The figures are given in feet and inches, and have been recalculated as centimeters here). The height of a northern male is given as 166 cm, and of five southern males, of which four were mounted skins and one a mounted skeleton, is 148–188 cm; of the four mounted skins, the smallest, from the Leiden Museum, has no associated skull or skeleton so that its maturity cannot be guaranteed (and its proportions seem peculiar compared to the others), and the next smallest is 175.3 cm. The height of a northern female is given as 160 cm; that of a southern female is 155.6 cm. Heller's length measurements are head-and-body, so not comparable to those of Hillman-Smith et al. [9].

Putting all these figures together, we get following body measurements:

Height [cm]

- Southern males 165–188 (n = 11), southern females 155.6–185 (n = 8)
- Northern males 151–165.7 (n = 3), northern females 150–160 (n = 6)

Length [cm]

- Southern males 259–284 (n = 10), southern females 248–273 (n = 4)
- Northern males 266–271 (n = 2), northern females 245–269 (n = 6)

Meagre as they are, these figures tend to substantiate the observation of Hillman-Smith et al. [9] that northern white rhinos are smaller – very markedly in the case of males, only slightly in the case of females. The spine length data are even more meagre, but appear to corroborate the height data. The weight discrepancies, however, are even greater for males than those for females about equivalent height.

It is possible to calculate height:length ratios from Heller [17]. For his northern sample, measured in the field, the range is 40.5–51.1% (n = 6); these are mostly immature, but the solitary value for an adult female falls squarely in the middle of the range. For an adult male, measured on a skeleton, we calculate 56.5%. For his southern sample, three males measured on mounted skins vary from 46.8–53.4%, one measured on a skeleton is 56.5%, and a female measured on a skeleton is 53.7%; the peculiar male, mentioned above, is only 41.9% (this specimen is of uncertain history and provenance; Jentink, [19], records only that it was brought to the Netherlands, date unstated, on the ship ‘Mauritius’ and presented by the Minister of Internal Affairs in 1879).

We attempted to measure height/body length proportions from photographs, but these are rather subjective. Impressionistically, and in agreement with Groves [1], we do tend to agree with Hillman-Smith [10] that northern white rhinos seem to stand higher in the leg than southern, which seem longer-bodied.

### Other External Features

**Figure 8:**
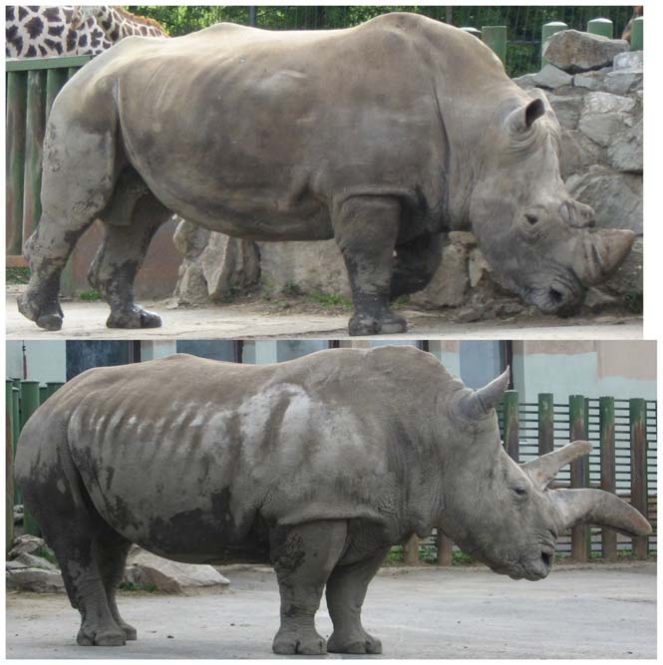
*Ceratotherium simum*. Left, Dan, male aged 40 years. Right, Zamba, female aged 37 years. Both, Usti nad Labem. Photos, Jan Robovsky.

**Figure 9:**
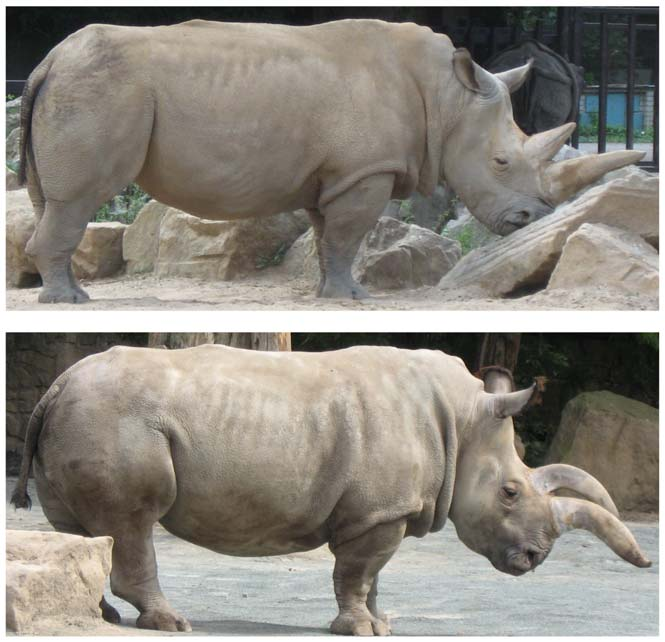
*Ceratotherium cottoni*. Left, Suni, male aged 27 years. Right, Nabire, female aged 24 years. Both, Dvur Kralove. Photos, Jan Robovsky.

On the basis of published photographs [20]–[27], and of observations and photographs by CPG of northern white rhinos in London, Antwerp, Dvur Kralove (also by JR) and San Diego, and of southern white rhinos in many institutions, there appear to be a number of consistent external differences between the two (See Figures 8, 9). Mostly, they concern the degree of skin folding and wrinkling, which deepens with age, and tends to be more marked in females than in males.

Costal grooving: no white rhino has the deep grooves, which correspond externally to the spaces between the ribs, which tend to characterise most black rhinos (*Diceros bicornis*). Some grooving nonetheless occurs in southern white rhinos, weak but becoming more accentuated with age, especially posteriorly, beginning about 2–3 ribs in front of the stifle fold. There is on the contrary little trace of costal grooving in northern white rhinos.

Fold over base of foreleg: if the animal is standing square, this fold always tends to be complete in southern white rhinos, but in northern it is usually less complete, not extending back to the elbow. This difference is not absolute.

Fold behind elbow: this is deep, fading upward toward the dorsal line, in southern white rhinos, but is hardly expressed in most northern ones.

Wrinkling around eye: southern white rhinos have deep circular wrinkles around the eye, but these are weak at the most in northern.

The dorsal profile is straighter in northern white rhinos, more concave behind the shoulder in southern.

The difference in the dorsal profile of the skull is readily appreciable on living animals.

Cave & Allbrook [28] could find no evidence of body hair in a subadult northern white rhino, whereas in southern white rhinos hairs were detectable by touch according to Alexander & Player [29]. The keepers at Dvur Kralove and Usti and Labem are of the opinion that this probably was a difference, although in southern whites they may become undetectable under insistent abrasion; and JR found no hairs on the flanks in three northern individuals (Saut [wild-born, died 2006], Najin and Suni [captive-born, still living]). CPG could detect no trace of body hairs by running a hand over the flanks of a docile northern white at San Diego Wildlife Park; hair was clearly detected by JR on the flanks of hybrid female Nasi, who died in 2007 in Dvur Kralove. This individual was bred under the same captive conditions as Saut, Najin and Suni and was older than Najin and Suni (if anything, hair would be expected to fall out, or at least abrade, with age). Note that hair is always to be found on the tail, muzzle at the base of the nasal horn, and ear rims, and (few, sparse) on the belly, throat, distal parts of both limbs, and apex of hump of both whites.

The keepers at Dvur Kralove Zoo are of the opinion that Northern white rhinos possess more shaggy ears and tail [30]. Several of the Northern white rhinos in Dvur Kralove Zoo are heavily shaggy on the ear rims (but some are not). We tend to consider these characters, based on observation of the many individuals of white and black rhinos in captivity, and wild as too variable for being diagnostic.

Fig. 8 depicts a male and female Southern White rhino; Fig. 9, a male and female Northern White rhino, but from zoos in the Czech republic. The horns have grown abnormally as a consequence of years of captivity.

### The Living Taxa: Behaviour and Ecology

Spassov [31] argued that the nuchal hump of the white rhinoceros serves the same function as the double horn: to enhance lateral visual display. The second horn duplicates the display function of the first horn, and the nuchal hump, which becomes apparent only in the head-up posture of lateral display, gives the impression of increased body size. One may take this further. In *Diceros bicornis*, the back is concave, leaving the withers and the croup as high points, whereas in *Ceratotherium*, both high points are duplicated, the withers by the nuchal hump, and the croup by the presacral eminence. The stimulus effect of the display is thereby increased.

The (former) distribution of the southern white rhino corresponded mostly to the Bushveldt Zone [32]. Northern white rhinos were said to live in open *Combretum* forest and nearby plains. According to Schomber [33], population densities in Umfolosi are notably higher than elsewhere in the range, and southern populations always seem to have existed at higher density than northern.

The social organisation of the southern white rhino was described in detail by Owen-Smith [18]. There are two types of mature males: territorial (or alpha) males, and non-territorial (or beta) males who do not reproduce and whose presence in an alpha male's territory is tolerated. Rachlow et al. [34] found that territorial males are on average older than non-territorial males, and though the same linear size (as measured by body length), are markedly larger in chest girth and neck girth. Only territorial males scent-mark by spray-urination and by scattering their faeces, kick with the hind legs before and after defecation, and beat the horns against bushes. They have much higher testosterone levels, and they consort much more with females of high reproductive value (non-pregnant females without calves less than 10 months old).

In a very small population of northern whites introduced to Murchison Falls National Park, van Gyseghem [27] recorded the same dominance behaviours displayed by the sole adult male: spray-urination, hindleg kicking, horn beating and displays towards subadult males. The home ranges of all the individuals of white rhinos in Murchison Falls National Park were found to be 5 to 10 times larger than those found in the southern subspecies in Hluhluwe Game Reserve [27].

At the “African rhino workshop, Cincinnati, October 1987” a discussion took place on possible behavioural/ecological differences between Northern and Southern white rhinos. It was reported that N. Owen-Smith noted that Southern white rhinos feed on short, nutritious grasses; given that the Northern white rhinos live in a wetter habitat, with long fibrous grasses, their feeding ecology could well differ, and K. Hillman-Smith concurred, but no research in Garamba had been conducted; her own casual observation indicated that Northern white rhinos “may eat more dicotyledons, and they have to survive in tall grasses such as *Hyparrhenia* and *Loudetia* in the wet season, and in burnt areas during the dry season. The social behaviour appears similar to that of the southern rhinos although ranges are about 10 times larger; which may be due to the very low population density in Garamba” [35].

The basic reproductive parameters (gestation, first oestrus, first copulation, mean oestrous cycle, receptivity), sperm morphology and social behaviour of Northern whites in captivity is similar or identical to Southern whites [36]–[39].

Policht et al. [40] confirmed that the repertoire of white rhino calls is much larger than that reported in other rhino species and also found an apparent similarity (large overlap) between acoustic parameters of homologous calls recorded in both forms of white rhinos.

Mikulica [36] observed a threat gesture involving swinging the head in Southern whites (in three individuals out of five), but not in Northern whites (six individuals). The behaviour was not noted by Backhaus [41] in Northern whites in the wild, but was detected in the captive population of Northern whites by Kuneš & Bičík [42] and Cinkova [43], but the latter did not observe it in Southern whites. This emphasises that rare behaviours may not be detected even with long observation periods (I. Cinkova, pers. comm., observed this behaviour only twice in 323 h of observation).

The single known hybrid between Northern and Southern white rhinos was Nasi, born 1977, and euthanasied in 2007 because of cancer and accompanying severe pain. Nasi's health seemed poor considering her age; we are unsure whether to attach any significance to this, but five older individuals (pure-bred Northern) are still living, born in 1972, 1973 and 1974).

The diploid chromosome number appears polymorphic in Northern white rhinos, as noted above [11]. Sudan (Studbook no. 372) had a diploid number of 81, and this character was inherited by his two female offspring, Nabire (No. 0789) and Najin (0943).

In conclusion, the reported behavioural and ecological observations on the Northern and Southern whites do not provide a clear taxonomic distinction between the two forms. Importantly, nor do they contradict such a distinction.

### Fossil white rhinos

Commonly, it has been assumed that, of the two African genera of rhinoceros, *Diceros*, with its browsing adaptations, is the more primitive, and can be traced back nearly unchanged to the Early Pliocene, for example at Laetoli, while the grazing *Ceratotherium* went through several evolutionary stages from the Early and Middle Pliocene *C. praecox* via the Late Pliocene/Early Pleistocene *C. simum germanoafricanum* to the modern white rhino [3]–[7]. Geraads [44] argued that it is in fact *Diceros* that has more derived skull shape, considering that the depth of the dorsal concavity increases during growth and the angle between the plane of the palate and the nuchal plane decreases in early ontogeny: the skull, in other words, becomes less like *Ceratotherium* with age. He transferred *C. praecox* to *Diceros*, and referred all the early stages of white rhinoceros to a species *Ceratotherium mauritanicum*, described from the Middle Pleistocene of North Africa (and surviving in North Africa until the Late Pleistocene, though replaced by *C. simum* in East Africa in the Early Pleistocene). The presumed stem species, from the Late Miocene of Greece and Iran, generally known as *Diceros neumayri*, he transferred to *Ceratotherium*, finding that though it was intermediate in cranial morphology, there were some respects (elongate antorbital portion of skull; occiput narrow base compared to crest) in which it more resembled modern *Ceratotherium*, and was a mixed feeder. He placed the separation of the two modern lineages “soon after the Miocene-Pliocene boundary”: *Diceros* evolved towards a browsing specialization, with smaller size, more transverse lophs on cheekteeth, more concave dorsal profile, while *Ceratotherium* became larger, with more inclined lophs and flatter dorsal profile. Kingston & Harrison [45], on the basis of stable isotope analysis of the teeth, attributed a mixed diet to rhinoceros from Laetoli (Middle Pliocene), which they referred to provisionally as *Ceratotherium praecox* (note that their reference to “modern Laetoli specimens” [45: pp. 288, 289] is an inadvertent error: the four modern specimens analysed for the paper — 1 from the Sudan, 1 from W. Madi in Uganda, 1 from Garamba in Zaire, and 1 from the Laikipia Plateau in Kenya — were inadvertently added to the sample of modern specimens of other large herbivores from Laetoli [John Kingston, personal communication to CPG]).

*Ceratotherium mauritanicum*, according to Geraads [44], differed from modern white rhinos by the weak postorbital constriction and wide nuchal crest, as well as the slender metapodials. He referred fossils from Kanapoi, Hadar, Dikika, Koobi Fora (below the KBS Tuff) and Rawi to it, but specimens throughout the Olduvai sequence were referred to *C. simum* (of which *germanoafricanum* was considered to be a synonym). Groves [1] had found that the type skull of *C. mauritanicum* and the skull from Koobi Fora were both wide postorbitally, and the Rawi skull fragment, like the Koobi Fora skull, had very broad occipital crests (that of the *C. mauritanicum* type skull is crushed in this region); skulls from Olduvai (both Bed II and Bed IV) resembled modern white rhinos in both these respects, but had extremely long toothrows, by which they differed from any modern white rhino.

### Genetics

The observed genetic divergences across taxa were consistent with the different evolutionary rates of the analyzed fragments. As expected, the nuclear fragment showed much lower levels of divergence due to the lower evolutionary rate of nuclear coding regions relative to mtDNA. Different evolutionary rates of the mitochondrial segments, especially the faster evolution of the D-loop, may explain the discrepancies in divergence observed by restriction digestion analysis of mtDNA in previous studies. Saturation consequent to its high evolutionary rate makes the D-loop unsuitable for analysis at the level of genera. The mitochondrial 12s, ND and the nuclear Amelogenin X fragments provide more meaningful generic comparisons.

The relative divergence of the presumed subspecies of white rhino was approximately twice (1.84±0.17) that between the black rhino subspecies, and was remarkably constant across all four fragments analysed. Assuming similar rates of divergence between the taxa, this argues for a longer separation of the white rhino taxa than the two black rhino taxa analyzed.

The divergence between the white rhino taxa as a percentage of the inter-generic divergence observed in previous studies were: for allozymes 1.6% [12], and for mtDNA 57% [15], 31% [14] and 15.4% [16]. With an observed divergence of 15.8% and 18.6%, for the ND and 12S fragments respectively, our results correspond well with that of Morales and Melnick [16]. Allozymatic analysis is generally not sensitive enough for assessing divergence at the level of subspecies. The higher rates observed by George et al. [14], [15] probably reflect a larger representation of the D-loop due to the particular restriction enzymes used. The divergence of 23.5% observed by us in respect of the Amelogenin fragment strongly supports the divergences observed in the 12S and ND segments. Due to the very low evolutionary rate of nuclear DNA, the estimate is based on only 1 and 2 mutations between the black rhino and white rhino taxa respectively, hence has lower resolving power than the mtDNA. Thus, we suggest 15–20% of the inter-generic divergence as a justifiable estimate of the divergence between the two white rhino taxa. The observed patterns of divergence and consistency of divergence ratios between taxa across analysed segments, justify the use of the estimated inter-generic divergence in assessing the divergence time of white rhino subspecies.

The *Ceratotherium* and *Diceros* divergence is dated to about 7 million years from the fossil record (Hooijer [3], although only the Miocene-Pliocene boundary according to Geraads [44]). The divergences observed by us in relation to the fossil evidence suggest a slower molecular clock in rhinos than in smaller mammals. Slow molecular clocks have been observed in elephants [46] and marine mammals [47] and maybe explained by the effects of longer generation time, increased body mass and lower metabolic rate on evolutionary rate [48]. Calibrating a molecular clock on the fossil evidence, the observed genetic divergence between the two white rhino taxa suggests their separation for at least 1–1.4 million years if Hooijer's [49] date for the separation of the two genera is correct, and 0.75–1 million years if Geraads' [44] date is more correct.

### The living taxa: taxonomy

**Figure 10:**
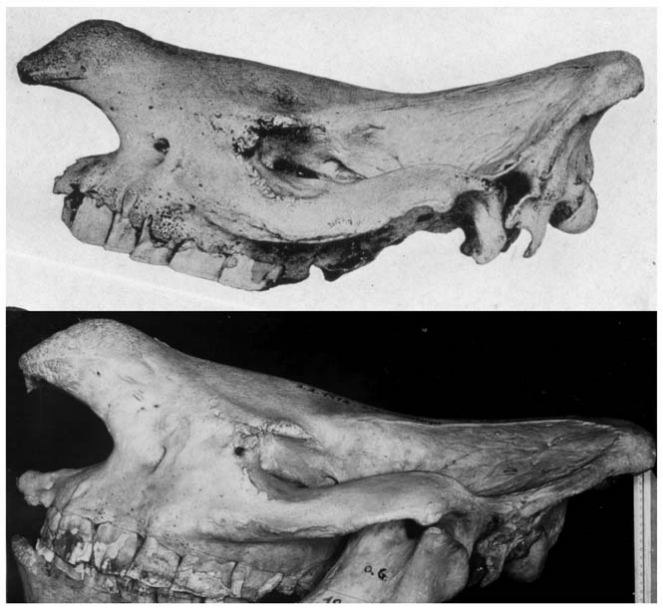
Skulls of adult males of *Ceratotherium simum* (above) and *C. cottoni* (below). Upper photo from Heller (1914, plate 17, fig. 3), lower photo by E. Trumler of skull in Zoologische Staatssammlung, Munich.

**Figure 11:**
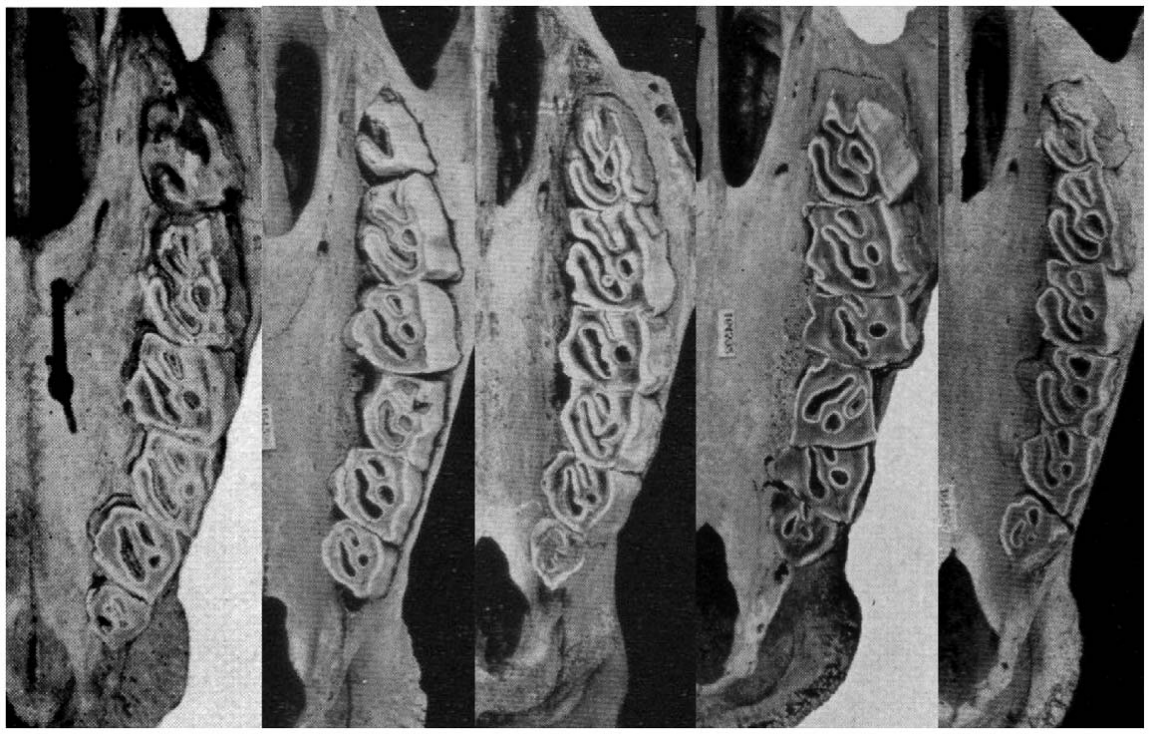
Maxillary toothrows of adult male *Ceratotherium simum* (left) and *C. cottoni* (second from left), and adult female *C.simum* (centre) and *C.cottoni* (second from right and right), from Heller (1913, plates 21:4, 21:2, 22:4, 21:1 and 22:3, respectively).

Northern and southern white rhinos have, without exception, been distinguished as subspecies within a single species, *Ceratotherium simum*. The northern form is universally distinguished as simply a subspecies, *Ceratotherium simum cottoni* (Lydekker, 1908), leaving the southern form as the nominotypical subspecies, *C. s. simum* (Burchell, 1821). We have, however, found that the two differ absolutely in numerous respects: the skull is readily distinguished (Figure 10), the dentition is somewhat different (Figure 11), they can be differentiated externally apparently without error, there is evidently a fixed difference in a serum enzyme and they are clearly distinguishable genetically in analysis of both mitochondrial and nuclear genomes. Under the Phylogenetic Species Concept (the only objective concept applicable to allopatric forms), we have no option but to consider them specifically distinct. While short separation times may characterise species pairs that are perfectly distinct by criteria of diagnosability and even reproductive isolation, a long time since separation does considerably strengthen other evidence for species status. Genetic analysis clearly indicates a separation time of over a million years between the two taxa, justifying their recognition as separate species: *Ceratotherium simum* (Burchell, 1821) and *Ceratotherium cottoni* (Lydekker, 1908).

### Conclusion

The northern white rhino is today on the verge of extinction. Its taxonomic distinctiveness argues strongly for its conservation, as its demise will mean the permanent loss of a unique taxon that is irreplaceable. The admirable success of the conservation histories of the Southern white rhino and the Indian rhino, both of which were brought back from the brink of extinction by successful conservation efforts, does, however, hold out hope that the northern white may yet be saved for posterity. With less than 20 individuals in the wild, the population cannot absorb any more poaching. It is very likely that any cause of increased mortality, of which poaching is the most threatening, and most easily addressed, will push them over the edge. Therefore, absolute protection from poaching is a must for the *in-situ* conservation of the species.

The highly successful management of white rhinos under semi-captive and captive conditions in Southern Africa indicates the importance of *ex-situ* conservation. Unlike in the case of the Javan rhino, where no captive population exists, and of the Sumatran rhino where captive breeding has only recently been achieved, and that only by a single female, the presence of a healthy if small captive population and their long history of successful management makes the *ex-situ* conservation of the northern white much more likely to be successful. For successful *in-situ* and *ex-situ* conservation of the northern white rhino, the lynch pin will be the availability of funding. In an age where billions of dollars are poured into saving companies going bankrupt and trillions into wars of arguable provenance, can we not spare a fraction of that to save a unique and charismatic megavertebrate and begin to address our disastrous impact on planet earth.

## Methods

### Morphometrics

CPG studied and measured skulls in the following collections (the abbreviations used here are in brackets following the names of the institutions): Natural History Museum, London (BM); Royal College of Surgeons, London (RCS); Powell Cotton Museum, Birchington (PC); Landesmuseum für Naturkunde, Karlsruhe (LNK); Zoologisches Institut, Hamburg (ZIH); Museum Royale de l'Afrique Centrale, Tervuren (MRAC); Naturalis, Leiden (RML); Naturhistoriska Riksmuseet, Stockholm (NRS); Muséum National d'Histoire Naturelle, Paris (NMP); American Museum of Natural History (AMNH); Smithsonian Institution (USNM).

The total number of skulls was 56 in all, the adult totals being as follows: Southern males 8, females 5; Northern males 18, females 14.

Fossil specimens are as follows (one specimen in each case): Tighenif (Ternifine), Algeria, latest Early Pleistocene or base of Middle Pleistocene, previously described by Arambourg [50]; measurements of a skull from Ileret (Koobi Fora Formation), Lake Turkana, Kenya, base of Early Pleistocene, taken from Harris [2]; Garusi, Tanzania, Middle Pliocene; Olduvai Bed II, Tanzania, Early Pleistocene; Olduvai Bed IV, Tanzania, early Middle Pleistocene; Kibish Formation (Omo River), Ethiopia, late Middle Pleistocene. Of these, only the first two (the Arambourg and Ileret skulls) are nearly complete; the others are fragmentary.

The following measurements were taken on each skull: Occipitonasal Length, Basal Length, Zygomatic Breadth, Occipital Breadth (occipital crest), Occipital Height (opisthion to opisthocranion), Nasal Breadth (nasal boss), Toothrow Length (P2 to M3), Depth of Dorsal Concavity (greatest distance from dorsal contour of cranium to a rod resting on nasal boss and occipital crest).

We entered measurements of skulls and teeth of white rhinos into a file in SPSS, version 12.0.1, and a made series of univariate and bivariate plots, and ran a series of discriminant analyses. Dental eruption stages follow a previous study [1].

JR studied hair distribution and took some body measurements on three adults of Southern whites (one male, two females), two immobilized adults of Northern whites (one male, one female), one dead male Northern white (not measured) and one euthanized hybrid female. Some standard measurements [9] were not always available (e.g. for time limitation in immobilized individuals) and sometimes they were difficult to obtain accurately (especially if the individuals were lying down). This was carried out in accord with the laws and ethical guidelines (No. 2045/2004-1020) established in the Czech Republic. Body measurements were approved by representatives of the Dvur Kralove ZOO (owner of the animals). The immobilization of measured individuals was carried out by representatives of the Dvur Kralove ZOO (with veterinary assistance) during the course of attempted artificial inseminations in collaboration with representatives of the Leibniz Institute for ZOO and Wildlife Research (Berlin). The procedure was noninvasive and did not involve any increased stress to the rhinos or increase in duration of the immobilisation.

### Genetics

Samples for genetic analysis consisted of blood or tissue. Except for those downloaded from GenBank, these were taken during the course of routine veterinary analysis by approved veterinary authorities of San Diego zoo; in no case did their extraction involve any increased stress to the rhinos or increase in duration of the immobilisation. Details of samples are given in Table 4. DNA extraction followed a phenol/chloroform extraction and QIAGEN column purification protocol. Primers and conditions for PCR amplification of 12S and D-loop mitochondrial fragments followed Fernando et al. [51]. The 12S primers amplified a 937 bp fragment of the mitochondrial 12S ribosomal RNA gene and the D-loop primers a 413 bp fragment incorporating 21 bp from the 3′ end of tRNA-Pro and 392 bp of the adjacent D-loop. Primers RH-ND-F, 5′-AAC AGT ACA ATT GAC TTC CAA 3′ and RH-ND-R, 5′ CCK GCG TTT AGT CGT TCT GTT 3′ for amplifying a mitochondrial NADH gene fragment were based on Indian rhino (Accession No. X97336) and white rhino (Accession No. NC001808) mtDNA sequences from GenBank. They amplified an approximately 1.2 kb fragment including part of tRNA-Glycine, NADH dehydrogenase subunit 3, tRNA-Arginine, NADH dehydrogenase subunit 4L and part of NADH dehydrogenase subunit 4. Primers Amel-3, 5′-GCA CCC TGG TTA TAT CAA CTT-3′ and Amel-6 5′-GGG TTC GTA ACC ATA GGA AG-3′ for amplification of the nuclear amelogenin (AMELX) gene were designed based on sequences from human, porcine and rat amelogenin (AMELX) genes. They amplified an approximately 1,685 bp fragment.

**Table 4:** Taxa and Origin of the Samples Used For Genetic Analysis.

Taxon	Country of origin	Locality of origin	Sample ID
*Ceratotherium simum simum*	South Africa	Kruger National Park	None (wild)
*Ceratotherium simum cottoni*	Zaire	Garamba National Park	San Diego Zoo NX# 28818
*Diceros bicornis michaeli* #1	Kenya	Solio Game Reserve, Naro-Moro	None (wild)
*Diceros bicornis michaeli* #2	Kenya	Captive born	Studbook No. 360
*Diceros bicornis minor* #1	South Africa	NA	None (wild)
*Diceros bicornis minor* #2	Zimbabwe	Zambezi Valley	None (wild)
*Diceros bicornis minor* #3	Zimbabwe	Zambezi Valley	None (wild)

Amplifications were conducted in ABI 9700 PCR thermocyclers, using 1 µl DNA extract, 18 µl PCR buffer dNTP mix, 0.5 µl 10 µM each primer, 0.1 µl *Taq* DNA polymerase, and 14.8 µl water. Amplifications were preceded by a 93°C step of 3 minutes. Samples were amplified for 40 cycles by denaturing at 93°C, annealing at 50°C and 66°C respectively for ND and Amelogenin primer pairs respectively, and extension at 72°C; each segment lasting one minute. Amplifications were followed by an extension step of 72°C for 15 minutes. Amplification products were sequenced in forward and reverse directions with the PCR primers and internal sequencing primers (ND-440, 5′-TTA CCA TAG CAC TAA TCC-3′; ND-310, 5′-CCA ATA GKA TCA GCA CGC CTA C-3′; ND-830, 5′-GTY ATR ATC TCC AAC ACT TAC-3′; and ND-920, 5′-CAC TAA CAT GAC TAT CAA-3′ for the ND fragment; AMEL328F 5′-CAT GAA ATA TAG ACT CGC TAA-5′, AMEL604F 5′-GCT CCT GCT CTT CTT TG-3′, AMEL1108F 5′-AAC AAT ATT TTG AAG TGT GGG-3′, and AMEL1116R 5′-TTA TAA TAC CCA CAC TTC AAA-3′for the Amelogenin X fragment). Sequences were edited, trimming ends with ambiguous peaks, and aligned with the program SEQUENCHER. Uncorrected p distance matrices were generated using the program PAUP* [52]. Sequences were deposited on GenBank (Table 5).

**Table 5:** GenBank Accession Numbers of Sequenced Fragments.

Taxon	D-loop	ND	12S	AMELX
*Ceratotherium simum simum*	AY742828	FJ608799	FJ608805	FJ608809
*Ceratotherium simum cottoni*	AY742829	FJ608800	FJ608806	FJ608810
*Diceros bicornis michaeli* #1	AY742830	FJ608801	-	FJ608811
*Diceros bicornis michaeli* #2	AY742831	FJ608802	FJ608807	-
*Diceros bicornis minor* #1	-	FJ608803	FJ608808	FJ608812
*Diceros bicornis minor* #2	AY742832	FJ608804	-	-
*Diceros bicornis minor* #3	AY742833	-	-	-
